# Supplementary material for: Health Literacy among Older Adults in Portugal and Associated Sociodemographic, Health and Healthcare-Related Factors
Source: Int J Environ Res Public Health. 2023 Feb 25;20(5):4172. doi: 10.3390/ijerph20054172 (PMC10002045; doi:10.3390/ijerph20054172)
Supplement: Supplementary file 1 [file ijerph-20-04172-s001.zip › ijerph-2221658-supplementary.pdf]

Table S1. HLS19-Q12 items and their categorisation according to domains and dimensions of health information processing.

HLS$_{19}$-Q12 item number	Health Literacy domain; Dimension of health information processing	Item wording On a scale from very easy to very difficult, how easy would you say it is:
1	1 - Healthcare;<br>A - Access/obtain information relevant to health	...to find out where to get professional help when you are ill?
2	1 - Healthcare;<br>B - Understand information relevant to health	...to understand information about what to do in a medical emergency?
3	1 - Healthcare;<br>C - Appraise/judge/evaluate information relevant to health	...to judge the advantages and disadvantages of different treatment options?
4	1 - Healthcare;<br>D - Apply/use information relevant to health	...to act on advice from your doctor or pharmacist?
5	2 - Disease prevention;<br>A - Access/obtain information relevant to health	...to find information on how to handle mental health problems?
6	2 - Disease prevention;<br>B - Understand information relevant to health	...to understand information about recommended health screenings or examinations?
7	2 - Disease prevention;<br>C - Appraise/judge/evaluate information relevant to health	...to judge if information on unhealthy habits, such as smoking, low physical activity or drinking too much alcohol, are reliable?
8	2 - Disease prevention;<br>D - Apply/use information relevant to health	...to decide how you can protect yourself from illness using information from the mass media?
9	3 - Health promotion;<br>A - Access/obtain information relevant to health	...to find information on healthy lifestyles such as physical exercise, healthy food or nutrition?
10	3 - Health promotion;<br>B - Understand information relevant to health	...to understand advice concerning your health from family or friends?
11	3 - Health promotion;<br>C - Appraise/judge/evaluate information relevant to health	...to judge how your housing conditions may affect your health and well-being?
12	3 - Health promotion;<br>D - Apply/use information relevant to health	...to make decisions to improve your health and well-being?

Adapted from: The HLS19 Consortium of the WHO Action Network M-POHL (2021): International Report on the Methodology, Results, and Recommendations of the European Health Literacy Population Survey 2019-2021 (HLS$_{19}$) of M-POHL. Austrian National Public Health Institute, Vienna. Retrieved from: https://m-pohl.net/sites/m-pohl.net/files/inline-files/HLS19%20International%20Report.pdf
